# Trem2-MICAL1-P-ERK Axis in Macrophages Confers Protection Against *Toxoplasma gondii*-Induced Adverse Pregnancy Outcomes

**DOI:** 10.3390/pathogens14111105

**Published:** 2025-10-30

**Authors:** Xiaoyu Geng, Haochen Yang, Zihan Wang, Ziqian Chen, Jinling Chen, Mei Yang

**Affiliations:** 1 Department of Pathogen Biology, School of Medicine, Nantong University, Nantong 226001, China; 2331310029@stmail.ntu.edu.cn (X.G.); yhcderek@163.com (H.Y.); ntwzh25@126.com (Z.W.); 16615176066@163.com (Z.C.); 2 Research Center of Clinical Medicine, Affiliated Hospital of Nantong University, Medical School of Nantong University, Nantong 226001, China

**Keywords:** *Toxoplasma gondii*, placental immunity, Trem2, soluble *T. gondii* antigens

## Abstract

*Toxoplasma gondii (T. gondii*) infection during pregnancy can cause severe placental damage and fetal impairment. Although triggering the receptor expressed on myeloid cells 2 (Trem2) confers protection against *T. gondii* infection, the precise molecular mechanisms underlying this immunoregulatory role remain incompletely understood. Using a mouse model, this study identifies a novel Trem2-MICAL1-P-ERK axis in macrophages that protects against *T. gondii*-induced adverse pregnancy outcomes (APO). RNA-seq of *Trem2*-overexpressing macrophages revealed significant upregulation of 1857 genes, with *MICAL1* among the most markedly altered, highlighting its potential role in Trem2-mediated signaling. Mechanistically, correlation analysis, molecular docking, fluorescence co-localization, and immunoprecipitation assays demonstrate that Trem2 directly interacts with MICAL1, which modulates downstream phosphorylated ERK (P-ERK) signaling. In a *T. gondii*-infected murine pregnancy model, genetic ablation of *Trem2* exacerbated pathogen-induced suppression of MICAL1 and P-ERK, whereas macrophage-specific overexpression of *Trem2*-*DAP12* restored this signaling axis. Conversely, *MICAL1* overexpression rescued P-ERK activation but failed to regulate Trem2 expression. Further studies in bone marrow-derived macrophages (BMDMs) revealed that *Trem2* deficiency potentiated the inhibitory effects of soluble *T. gondii* antigens (*Tg*Ag) on MICAL1 and P-ERK. These findings elucidate how *T. gondii* disrupts placental immunity through targeted suppression of Trem2-mediated signaling and establish the Trem2-MICAL1-P-ERK cascade as a core regulatory pathway in immune homeostasis during pregnancy.

## 1. Introduction

*T. gondii* infection is a globally distributed zoonotic disease [1]. When contracted during pregnancy, *T. gondii* can cross the placental barrier, causing congenital toxoplasmosis, manifesting as fetal malformations, intellectual disabilities, and various pregnancy complications [2]. Studies demonstrate that *T. gondii* induces APO mainly by disrupting the homeostasis of the maternal–fetal immune microenvironment [3,4]. The maternal–fetal interface is a highly dynamic and multicellular collaborative system that serves as the critical region for immune interactions between mother and fetus during pregnancy [5,6]. Composed of maternal decidual immune cells and placental trophoblasts, it regulates and maintains pregnancy immune tolerance [7]. *T. gondii* can manipulate the expression of inhibitory molecules (e.g., LILRB4, B7-H4, and Tim-3) on decidual immune cells, leading to immune dysfunction and APO [8,9,10]. Decidual immune cells primarily include decidual natural killer (dNK) cells, decidual macrophages (dMφ), and decidual dendritic cells [11]. As the second most abundant immune cell population at the maternal–fetal interface, dMφ play essential roles in maintaining immune homeostasis and clearing apoptotic cells—physiological processes crucial for healthy pregnancy [12]. In *T. gondii*-induced APO, dMφ dysfunction is primarily mediated through the regulation of key signaling pathways and effector molecules [13]. Research shows that *T. gondii*-secreted effector proteins (e.g., ROP16 and GRA15) interfere with normal dMφ polarization by activating the STAT3/STAT6 signaling pathway [13,14,15,16]. Additionally, RNA-Seq data confirm that *T. gondii* infection upregulates pro-inflammatory genes while reducing F-actin polymerization and planar cell polarity, thereby destabilizing the host cytoskeleton [17]. Given these findings, this study investigates the key effector molecules and signaling pathways in dMφ that mediate immune imbalance following *T. gondii* infection, aiming to elucidate the molecular mechanisms by which the pathogen disrupts maternal–fetal interface homeostasis.

Recent studies reveal that Trem2, specifically expressed in dMφ, plays a regulatory role in this process [18]. As a pivotal immune receptor in phagocytes, Trem2 directly mediates clearance of invasive pathogens by modulating reactive oxygen species (ROS) generation [19]. Its overexpression enhances chemokine receptor expression, promotes cellular migration, and augments phagocytic capacity, whereas endogenous Trem2 downregulation not only suppresses phagocytosis but also significantly increases transcription of pro-inflammatory cytokines (e.g., TNF-α and IL-1β) [20]. The execution of phagocytosis fundamentally relies on dynamic cytoskeletal reorganization [21]. MICAL1, an actin oxidase, mediates F-actin depolymerization, a critical step in cytoskeletal remodeling [22]. *T. gondii* surface antigen *Tg*SAG1 has been shown to disrupt host S100A6–vimentin complexes, thereby inhibiting actin polymerization while simultaneously promoting TNF-α secretion via the S100A6–Vimentin/PKCθ-NF-κB signaling pathway [23,24]. Beyond its impact on cytoskeletal regulation, *T. gondii* also modulates host transcriptional programs: our recent work revealed that *T. gondii* suppresses Trem2 promoter activity via the transcription factor ATF3, a mechanism linked to APO [25]. However, the downstream pathways through which Trem2 exerts its protective effects remain to be fully elucidated. Notably, studies demonstrate that MICAL1 depletion suppresses P-ERK expression, while its overexpression enhances P-ERK nuclear translocation, highlighting its crucial role in ERK signaling activation [26].

To validate the protective role of the Trem2-MICAL1-P-ERK pathway, we established *T. gondii*-infected pregnancy models using both wild-type (WT) and *Trem2*^−/−^ mice. Our results revealed that *T. gondii* infection significantly downregulates Trem2 expression and suppresses MICAL1-P-ERK signaling activity. Notably, Trem2 deficiency significantly exacerbated the suppression of MICAL1-P-ERK signaling activity upon *T. gondii* challenge. These findings collectively indicate that *T. gondii* disrupts placental immune defense by targeting the Trem2-MICAL1-P-ERK signaling axis. This study aims to elucidate the precise molecular mechanisms underlying this pathogenic process, potentially offering novel therapeutic strategies for *T. gondii* infection-induced pregnancy complications.

## 2. Materials and Methods

### 2.1. Mice

C57BL/6 mice were housed under standard conditions at Nantong University’s animal facility. *Trem2*-deficient mice (B6/JGpt-*Trem2* em1Cd3332in1/Gpt knockout) were created through CRISPR/Cas9-mediated gene editing by Gempharmatech (Nanjing, China). The experimental mice were randomly assigned to either the WT or *Trem2*^−/−^ group. Each group included 8 male and 16 female mice, all weighing between 25 and 30 g. To establish mating pairs, one male mouse was co-housed with two female mice per cage. An investigator, who was blinded to the experimental groups, performed all allocations using a random number table. Successful mating was confirmed by vaginal plug detection before 8:00 a.m., with plug-positive females designated as gestation day 0.5 (GD0.5) [27]. The pregnant WT or *Trem2*^−/−^ mice were randomly allocated to either the normal pregnancy (NP) group or the *T. gondii* infection (TI) group, which received 300 tachyzoites (RH strain) via intraperitoneal injection at GD8.5. On GD17.5, CO_2_ euthanasia was performed, followed by placental collection for subsequent experiments. All pregnant mice were monitored continuously, and their survival was ensured throughout the study. Accordingly, no unexpected lethal outcomes were observed during the in vivo experiments. All mouse procedures received approval from the Animal Care and Use Committee of Nantong University (protocol #P20230302–013).

### 2.2. Preparation of T. gondii Antigens

The antigens were extracted from *T. gondii* tachyzoites (RH strain) [28]. Briefly, 1 × 10^8^ viable tachyzoites (>95% viability by trypan blue) were incubated in serum-free RPMI-1640 medium for 3 h at 37 °C. The culture supernatant was collected and concentrated with Amicon^®^ Ultra-15 centrifugal filters (Merck Millipore, Darmstadt, Germany). Endotoxins were subsequently removed by an endotoxin removal kit (Thermo Fisher Scientific, Waltham, MA, USA). Aliquots were stored at −80 °C until use.

### 2.3. Cell Culture

The murine macrophage cell line (Raw264.7 cell) was ordered from the National Collection of Authenticated Cell Cultures (Shanghai, China) and maintained in complete DMEM (Thermo) containing 10% heat-inactivated FBS (ExCell Bio, Suzhou, China) and 1% penicillin-streptomycin (Thermo). Raw264.7 cells were incubated at 37 °C in a 5% CO_2_ humidified atmosphere and subcultured when they reached 70–80% confluency. For experimental treatments, cells were stimulated with *Tg*Ag (5 µg/mL) for 48 h. For primary macrophage isolation, BMDMs were prepared from WT mice (6–8 weeks old) and *Trem2*^−/−^ mice (6–8 weeks old). Briefly, femurs and tibias were aseptically dissected and flushed with cold PBS, then transferred to complete medium for immersion. Bone ends were opened with sterile scissors, and marrow was flushed using complete medium through a syringe until the bones appeared white. Cells were treated with 5 volumes of red blood cell lysing buffer, centrifuged, and resuspended in DMEM before filtering through a 200-μm mesh. Isolated bone marrow cells were cultured in complete medium containing murine M-CSF (20 ng/mL). After 6 days of differentiation, mature macrophages were harvested on day 8 for subsequent experiments.

### 2.4. RNA Quantification and Sequencing

Total RNA was extracted from murine macrophage Raw264.7 cells, using Trizol reagent (Invitrogen, Carlsbad, CA, USA) according to the manufacturer’s instructions. RNA integrity and concentration were verified using an Agilent 2100 Bioanalyzer (Agilent Technologies, Santa Clara, CA, USA) and agarose gel electrophoresis. RNA library preparation and sequencing of Raw264.7 cells were performed at Guangzhou GeneDenovo Biotechnology Co., Ltd. (Guangzhou, China). Briefly, poly(A) + mRNA was enriched from total RNA using Oligo (dT) magnetic beads. The purified mRNA was fragmented into short fragments (200–700 nucleotides), followed by first-and second-strand cDNA synthesis using random hexamer primers. The double-stranded cDNA was end-repaired, adenylated, and ligated with Illumina (San Diego, CA, USA) sequencing adapters. The adapter-ligated fragments were then purified and enriched by PCR amplification to generate the final sequencing library. Library quality was assessed using an Agilent High Sensitivity DNA Kit, and qualified libraries were subjected to paired-end sequencing (PE150) on an Illumina NovaSeq 6000 platform.

### 2.5. Immunoblotting

For protein analysis, cells or tissues were lysed in RIPA buffer containing protease inhibitors as previously described [29]. After quantifying protein concentration, equal amounts were separated by SDS-PAGE (8–12%) and transferred into PVDF membranes (Merck Millipore). PVDF membranes were blocked with 5% non-fat milk buffer for 1 h at room temperature (RT) and then incubated overnight at 4 °C with primary antibodies: Trem2 mouse antibody (R&D Systems, 1: 3000, #AF1729, Minneapolis, MN, USA); MICAL1 rabbit polyclonal antibody (Proteintech, 1: 3000, #14818–1-AP, Rosemont, IL, USA), ERK1/2 rabbit polyAb (Proteintech, 1: 3000, #11257–1-AP), P-ERK1/2 rabbit mAb (Cell Signaling Technology (CST), 1: 3000, #4370, Danvers, MA, USA), GAPDH mouse monoclonal antibody (CST, 1:50,000, 1E6D9, #60004–1-Ig). Following TBST washes, membranes were probed with HRP-conjugated goat anti-mouse IgG (H + L) (Proteintech, 1: 5000, #SA00001–1) or HRP-conjugated goat anti-rabbit IgG (H + L) (Biosharp, 1: 5000, #BL003A, Nantong, China) secondary antibodies for 1 h at RT. Protein band was visualized using ECL substrate (Meilunbio, Dalian, China) and analyzed with ImageJ software (version 4.0.1), with GAPDH serving as loading controls.

### 2.6. Immunofluorescence Staining

Cells on coverslips were fixed with 4% paraformaldehyde for 15 min, permeabilized with 0.1% Triton X-100 for 5 min, and blocked with 5% BSA for 10–30 min. Primary antibodies were incubated for 1 h at RT: recombinant anti-Trem2 rabbit antibody (Abcam, 1:500, EPR26210–1, #ab305103, Cambridge, UK), MICAL1 rabbit Polyclonal antibody (Proteintech, 1:200, #14818–1-AP), ERK1/2 rabbit polyAb (Proteintech, 1: 10, #11257–1-AP), P-ERK1/2 rabbit mAb (CST, 1:200, #4370), followed by incubation with fluorophore-conjugated secondary antibodies for 10 min. Signal amplification solution was applied for 10 min, and nuclei were counterstained with DapI for 5 min (Absin, Shanghai, China). Coverslips were mounted and imaged using confocal microscopy.

### 2.7. Co-Immunoprecipitation

The cell lysates were prepared using RIPA buffer supplemented with PMSF. Following the manufacturer’s protocol, 2 μg of primary antibody was introduced to the supernatant and incubated at 4 °C for 2 h. Subsequently, Protein A/G Plus-Agarose (SC-2003, Santa Cruz Biotechnology, Dallas, TX, USA) was added, and the mixture was rotated overnight at 4 °C. After centrifugation, the immunoprecipitated complexes were collected and washed repeatedly with lysis buffer. Finally, both the precipitated samples and input controls were subjected to a Western blot for the detection of co-precipitated proteins.

### 2.8. Overexpression Constructs

The *Trem2-DAP12* fusion was designed by linking the extracellular domain of mouse Trem2 (aa19–171) to mouse DAP12 (aa28–114). A D52A mutation was introduced in DAP12 to prevent binding with other receptors, enabling stable cell surface expression of the *Trem2*-*DAP12* complex. The native signal peptide was replaced with the mouse immunoglobulin κ light chain leader sequence, and a FLAG tag was added at the N-terminus of Trem2 for detection [30]. The lentiviral vectors overexpressing *Trem2*/*MICAL1* (pcSLenti-EF1-EGFP-F2A-Puro-CMV-Igκ leader-3xFLAG-*Trem2* (19–171aa)-*Tyrobp* (28–114aa, G52A)-WPRE/pcSLenti-EF1-EGFP-P2A-Puro-CMV-*MICAL1*-3xFLAG-WPRE) and control empty vectors (pcSLenti-EF1-EGFP-F2A-Puro-CMV-MCS-WPRE/pcSLenti-EF1-EGFP-P2A-Puro-CMV-MCS-3xFLAG-WPRE) were packaged by OBiO Technology (Shanghai, China). Raw 264.7 cells at 30–40% confluency were infected with lentivirus at MOI = 40. After 12–16 h, the medium was replaced with fresh complete DMEM. Selection began at 72 h post-infection using 2 µg/mL puromycin (Beyotime, Shanghai, China) for 7 days. Overexpression efficiency was validated by Western blot.

### 2.9. Statistical Analysis

All data represent at least three independent experimental replicates and are presented as mean ± standard deviation (SD). Statistical comparisons were performed using GraphPad Prism 9.0 (La Jolla, CA, USA). For two-group comparisons, we applied an unpaired Student’s *t*-test. Multiple group analyses employed either one-way ANOVA followed by Tukey’s post hoc test (for single-variable experiments) or two-way ANOVA with Sidak’s multiple comparisons test (for factorial designs). In all analyses, probability values (*p*) below 0.05 were considered statistically significant.

## 3. Results

### 3.1. T. gondii-Induced Downregulation of Trem2 Expression

**Figure 1:**
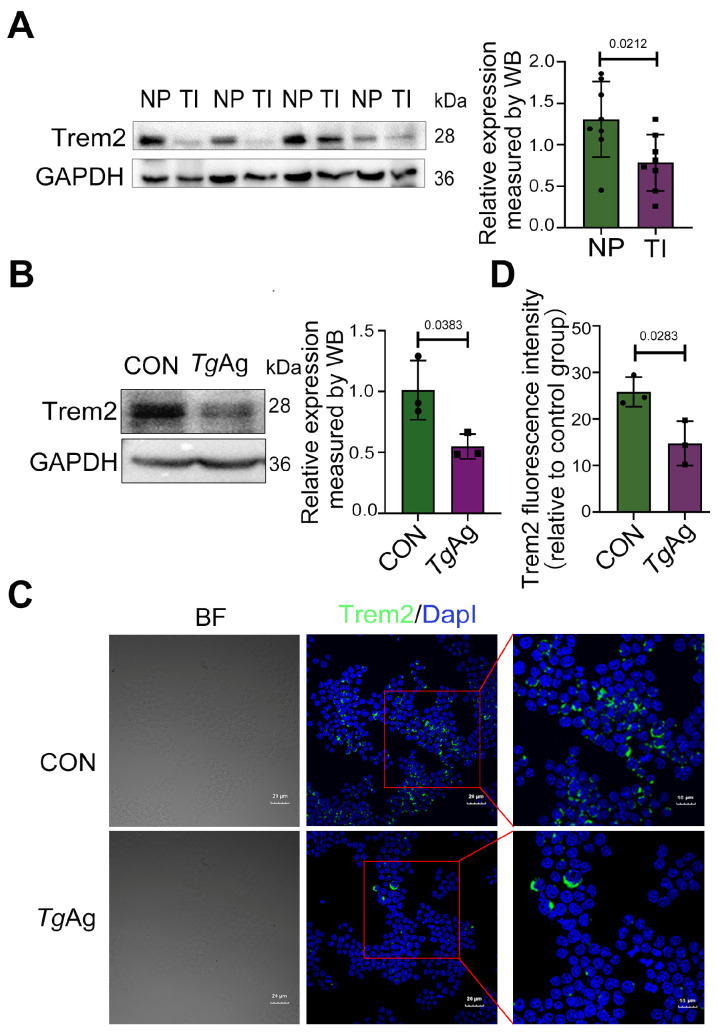
*T. gondii*-induced downregulation of Trem2 expression. (**A**) Placental tissues were collected from WT mice with NP or TI at GD 17.5. Protein levels were analyzed by WB (n = 8 mice per group). (**B**) WB analysis of Raw264.7 cells after 48 h exposure to *Tg*Ag (5 μg/mL). (**C**) Immunofluorescence staining of Raw264.7 cells showing Trem2 (green) and DapI (blue) following 48 h treatment with *Tg*Ag (5 μg/mL). (**D**) Quantitative analysis of Trem2 fluorescence intensity from panel C. Scale bars, 20 µm. NP: Normal pregnancy; TI: *T. gondii* infection; CON: Untreated control group; *Tg*Ag: Soluble *T. gondii* antigens; n = 3 per group; A two-tailed unpaired Student’s *t*-test was used (**A**,**B**,**D**). Western blot bands were quantified using ImageJ, normalized to GAPDH.

As Trem2 regulates key immunological processes at the maternal–fetal interface and modulates phagocyte activity [31], we investigated its expression changes during *T. gondii* infection. Pregnant mice, intraperitoneally injected with *T. gondii* tachyzoites at GD8.5, displayed a significant downregulation of Trem2 in placental tissues collected at GD17.5 compared to non-infected controls, as evidenced by Western blot analysis (Figure 1A). Trem2 expression was primarily observed in macrophages [20]. Therefore, in our in vitro experiments, we employed Raw264.7 cells (the murine monocyte macrophage leukemia cell line) to observe the role of *Tg*Ag on Trem2 expression. We found that the treatment of Raw264.7 cells with 5 µg/mL *Tg*Ag substantially decreased Trem2 level relative to untreated cells (CON) (Figure 1B), which was consistently observed in in vivo experiments. Immunofluorescence analysis confirmed these findings, demonstrating markedly reduced Trem2 signal intensity (green) in *Tg*Ag-exposed cells versus controls, with DapI (blue) counterstaining identifying nuclear localization (Figure 1C,D). These data collectively establish that *T. gondii* mediates Trem2 suppression in both placental tissues and macrophages.

### 3.2. T. gondii Disrupts Trem2-MICAL1 Molecular Interplay in Macrophages

Existing research has established that *T*. *gondii* infection induces macrophage dysfunction and compromises trophoblast function as well as placental development, whereas Trem2 upregulation can counteract these detrimental effects [18]. To elucidate the molecular mechanisms underlying Trem2-mediated protection, we aimed to identify potential Trem2-interacting molecules in macrophages involved in the host response to *T. gondii* infection.

**Figure 2:**
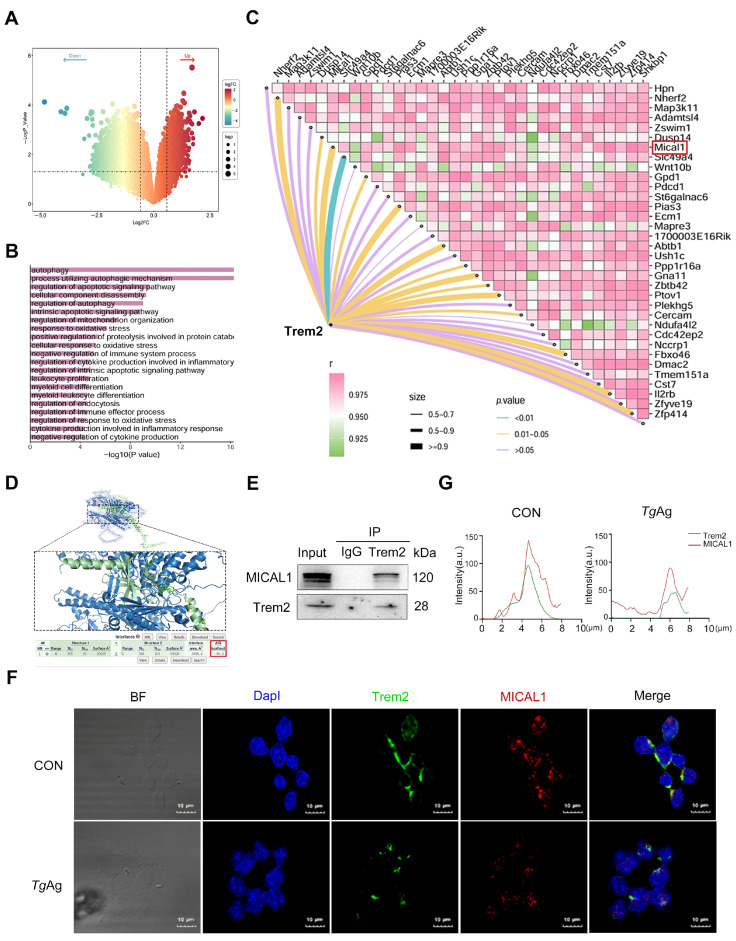
*T. gondii* disrupts Trem2-MICAL1 molecular interplay in macrophages. (**A**) Volcano plots of differentially expressed genes, screening criteria: |log_2_ (fold-change) (FC)| > 1.5, *p* Value < 0.05, Right-side genes are upregulated in *Trem2*-overexpressing macrophages, while left-side genes are downregulated. (**B**) GO biological process enrichment analysis of differentially expressed genes in *Trem2*-overexpressing macrophages. (**C**) Correlation matrix analysis between the hub gene *Trem2* and various differentially expressed genes. (**D**) Protein-protein docking was performed using PDBePISA, which predicted a high-affinity binding between Trem2 and MICAL1 with a binding energy of −36.2 kcal/mol. Structural visualization of the Trem2-MICAL1 complex was generated using Pymol. (**E**) Cell lysates were immunoprecipitated with anti-Trem2 or control IgG antibody, followed by immunoblotting with antibodies against MICAL1 and Trem2. (**F**) Immunofluorescence analysis demonstrating double co-localization of Trem2 (green) and MICAL1 (red) in Raw264.7 cells. Compared to the CON, *Tg*Ag-stimulated (5 μg/mL, 48 h) groups exhibit reduced co-localization. Nuclei were counterstained with DapI (blue). (**G**) Fluorescence intensity and co-localization analysis of Trem2 and MICAL1 in *Tg*Ag-stimulated Raw264.7 macrophages were performed using ImageJ. Data are presented in arbitrary units (a.u.). CON: Untreated control group; *Tg*Ag: Soluble *T. gondii* antigens.

Through RNA-seq analysis of *Trem2*-overexpressing Raw264.7 macrophages, we identified a total of 1857 significantly up-regulated differentially expressed genes (screening criteria: |log_2_ (fold-change) (FC)| > 1.5, *p* Value < 0.05) (Figure 2A). Among these, *MICAL1* was ranked among the top 50 up-regulated genes. To further elucidate the functional implications of *Trem2* overexpression, Gene Ontology (GO) enrichment analysis revealed that the upregulated genes were significantly associated with autophagy-related processes (Figure 2B). Previous studies demonstrated that Trem2 expression can be upregulated through downregulation of ANGPTL2, leading to effective enhancement of autophagy [32,33]. Correlation analysis of differentially expressed genes further highlighted *MICAL1* as one of the most strongly positively correlated genes with the hub gene Trem2 (Pearson R ≥ 0.9) (Figure 2C). Given that MICAL1 is a known regulator of cytoskeletal dynamics, and its knockdown has been shown to suppress autophagy (reducing Beclin1 and LC3B) and promote apoptosis (increasing Bax and C-caspase 3), we propose that Trem2 may enhance macrophage phagocytosis and autophagy by upregulating MICAL1 to facilitate cytoskeletal remodeling [34,35,36].

Molecular docking analysis using PDBePISA and Pymol predicted that Trem2 and MICAL1 may potentially bind to each other (Figure 2D). The docking results revealed a strong binding affinity with a binding energy of −36.2 kcal/mol. To experimentally validate this interaction, co-immunoprecipitation (co-IP) assays were conducted. The results showed that Trem2 specifically immunoprecipitated with MICAL1 in macrophages, confirming their direct molecular association (Figure 2E). The immunofluorescence co-localization experiments confirmed significant basal co-localization of Trem2 and MICAL1 in unstimulated macrophages. Following stimulation with *Tg*Ag, we observed significant downregulation in the expression levels of both Trem2 and MICAL1, along with a marked reduction in their co-localization, indicating disruption of their interaction (Figure 2F,G). These findings collectively suggest that the disruption of the Trem2-MICAL1 interaction may represent a key molecular mechanism by which *T. gondii* impairs macrophage phagocytic function and immune evasion.

### 3.3. T. gondii Suppresses Both MICAL1 and P-ERK Expression

**Figure 3:**
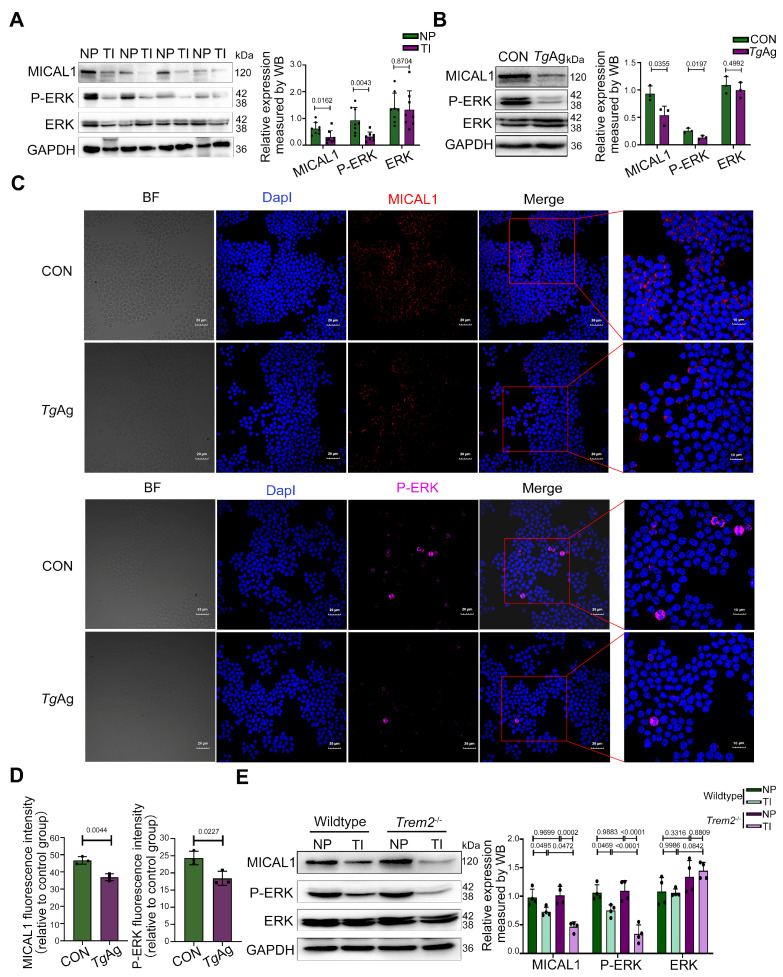
*T. gondii* suppresses both MICAL1 and P-ERK expression. (**A**) WB analysis of MICAL1, P-ERK, and total ERK expression in placental tissues from NP and TI groups in wildtype mice at GD17.5 (n = 8 mice per group). (**B**) WB analysis of MICAL1, P-ERK, and total ERK in cells stimulated with or without *Tg*Ag (5 μg/mL, 48 h). n = 3 per group; (**C**) Immunofluorescence staining showing MICAL1 (red) and P-ERK (magenta) localization in Raw264.7 cells under CON and *Tg*Ag (5 μg/mL, 48 h)-stimulated conditions. Nuclei were counterstained with DapI (blue). (**D**) Quantitative analysis of fluorescence intensity for MICAL1 and P-ERK in *Tg*Ag-treated Raw264.7 cells. n = 3 per group; (**E**) WB analysis of MICAL1, P-ERK, and total ERK expression in placental tissues from WT and *Trem2*^−/−^ mice (NP and TI groups) at GD17.5. n = 4 mice per group; NP: Normal pregnancy; TI: *T. gondii* infection; CON: Untreated control group; *Tg*Ag: Soluble *T. gondii* antigens; Performed statistical analysis using a two-tailed unpaired Student’s *t*-test (**A**,**B**,**D**) and two-way ANOVA with Sidak′s multiple comparisons test (**E**). Western blot bands were quantified using ImageJ, normalized to GAPDH.

Extending Trem2’s established functions in phagocyte regulation and signal transduction, we provide the first evidence whereby *T. gondii* coordinately downregulates the expression of the Trem2, MICAL1, and P-ERK axis in mouse placentas (Figure 3A). This suggests that the suppression of P-ERK may be a consequence of MICAL1 downregulation, consistent with the known functional link between MICAL1 and ERK activation [26]. This suppression was recapitulated in vitro, where *Tg*Ag-stimulated Raw264.7 macrophages exhibited reduced protein levels of Trem2, MICAL1, and P-ERK (Figure 3B). Similarly, immunofluorescence analysis further revealed that *Tg*Ag stimulation markedly decreased the fluorescence intensity of MICAL1 and P-ERK in macrophages (Figure 3C), while total ERK levels remained unchanged. To establish that Trem2 is essential for MICAL1 and P-ERK signal transduction during *T. gondii* challenge, we performed immunoblotting to quantify MICAL1, P-ERK, and ERK levels in *Trem2*^−/−^ mice. Quantitative comparisons revealed comparable MICAL1 and P-ERK expression between uninfected *Trem2*-deficient and WT placental tissues. However, infected *Trem2* knockout mice showed substantially lower MICAL1 and P-ERK levels in placental tissue relative to infected WT controls (Figure 3D). We hypothesize that compensatory upregulation of other receptors or signaling pathways may maintain P-ERK and MICAL1 expression under basal conditions. Nevertheless, the enhanced suppression of MICAL1 and P-ERK upon the infection in *Trem2*^−/−^ mice indicates that Trem2 likely plays a critical protective role during the infection, alleviating the parasite’s ability to over-suppress MICAL1/P-ERK signaling.

### 3.4. Functional Rescue of MICAL1/P-ERK Pathway by Trem2 in T. gondii-Infected Macrophage

**Figure 4:**
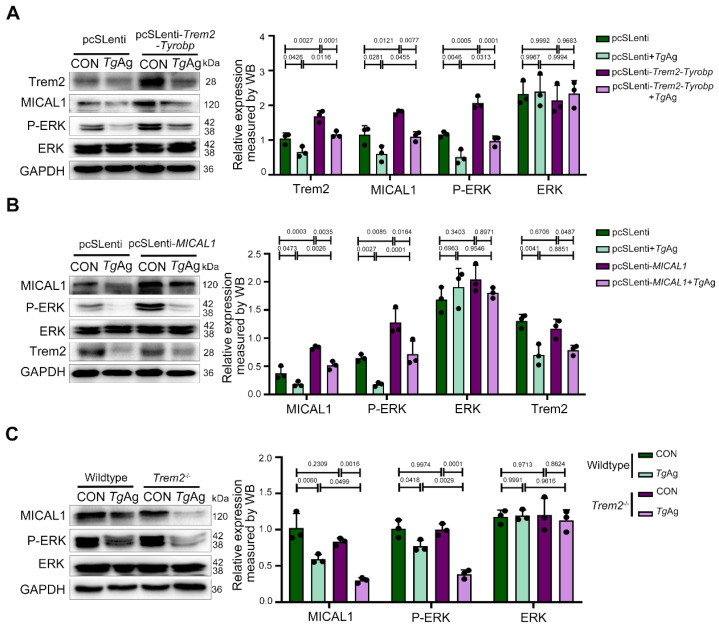
Functional rescue of MICAL1/P-ERK pathway by Trem2 in *T. gondii* antigen-stimulated macrophage. (**A**) Overexpression of *Trem2-Tyrobp* in Raw264.7 cells: WB analysis of Trem2, MICAL1, P-ERK, and total ERK expression in pcSLenti-empty vector (control) and pcSLenti-*Trem2*-*Tyrobp* transfected cells, with or without *Tg*Ag (5 μg/mL, 48 h) treatment. (**B**) Overexpression of *MICAL1* in Raw264.7 cells: WB analysis of MICAL1, P-ERK, total ERK, and Trem2 expression in pcSLenti-empty vector and pcSLenti-*MICAL1*-transfected Raw264.7 cells, with or without *Tg*Ag stimulation (5 μg/mL, 48 h). (**C**) WB analysis of MICAL1, P-ERK, and total ERK expression in BMDMs from wildtype and *Trem2*^−/−^ mice (CON and *Tg*Ag). CON: Untreated control group; *Tg*Ag: Soluble *T. gondii* antigens; n = 3 per group; Statistical analysis was performed using two-way ANOVA with Sidak′s multiple comparisons test (**A**–**C**). Western blot bands were quantified using ImageJ, normalized to GAPDH.

To confirm the role of Trem2 in regulating the MICAL1/P-ERK signaling pathway during *T. gondii* infection, we introduced a *Trem2-DAP12* fusion construct into Raw264.7 cells that maintains native Trem2 signaling through DAP12. The chimeric protein was generated by fusing the extracellular and transmembrane domains of mouse *Trem2* (aa19–171) with the transmembrane and cytoplasmic regions of mouse *DAP12* (aa28–114), incorporating a G52A mutation in *DAP12* to prevent interactions with other receptors and ensure proper formation of the *Trem2-DAP12* complex [30]. Four experimental groups were established: pcSLenti (empty vector control), pcSLenti + *Tg*Ag, pcSLenti-*Trem2*-*Tyrobp* (*Trem2*-*DAP12* complex overexpression), and pcSLenti-*Trem2*-*Tyrobp* + *Tg*Ag. Significantly elevated Trem2 expression in the pcSLenti-*Trem2*-*Tyrobp* group confirmed successful establishment of the *Trem2*-*DAP12* overexpression model. Compared to control macrophages carrying the pcSLenti, *Tg*Ag stimulation caused significant downregulation of MICAL1 and P-ERK expression (Figure 4A). Crucially, this suppression was completely reversed in cells overexpressing the *Trem2*-*Tyrobp* complex, with protein levels restored to baseline. These results demonstrate that *Trem2*-*DAP12* signaling is sufficient to protect the MICAL1/P-ERK pathway from pathogen-mediated disruption. We next stably overexpressed *MICAL1* in Raw264.7 macrophages, generating four groups: pcSLenti, pcSLenti + *Tg*Ag, pcSLenti-*MICAL1* (*MICAL1* overexpression), and pcSLenti-*MICAL1* + *Tg*Ag. Elevated MICAL1 expression in pcSLenti-*MICAL1* macrophages confirmed successful model generation. Compared to the control, *Tg*Ag induced significant downregulation of Trem2 and P-ERK. While *MICAL1* overexpression significantly attenuated the suppression of P-ERK, it notably did not reverse the *Tg*Ag-induced downregulation of Trem2 (Figure 4B). These findings indicate that although MICAL1 functions downstream to modulate ERK activity, it was unable to compensate for the Trem2 deficiency during infection. Consistent with our observations in placental tissue, studies in BMDMs from *Trem2*^−/−^ mice revealed no difference in MICAL1 or P-ERK expression compared to WT BMDMs under uninfected conditions. However, following *Tg*Ag stimulation, the suppression of MICAL1 and P-ERK was significantly exacerbated in *Trem2*^−/−^ BMDMs compared to stimulated WT controls (Figure 4C). The concordance between placental tissue protein data and BMDM results solidifies Trem2’s status as a master regulator of anti-*T. gondii* defense in macrophages, highlighting its potential as a therapeutic target for protecting MICAL1/P-ERK signaling from pathogen assault.

## 4. Discussion

The effective control of toxoplasmosis, a globally distributed zoonosis caused by *T. gondii*, necessitates a One Health approach [37]. Central to this control is the strengthening of coordination, collaboration, and communication at the human–animal–environment interface, a measure fundamental to ensuring global health security [38]. Within the host (including humans and animals), *T. gondii* promotes its own survival and replication by modulating host cell function, signal transduction, and immune responses [39]. The immunosuppressive conditions during pregnancy make pregnant women more susceptible to *T. gondii*, and this susceptibility is closely linked to the immune characteristics of the maternal–fetal interface [40]. At this interface, dMφ serves as a crucial immune barrier, playing a central protective role in maintaining pregnancy homeostasis [41]. However, *T. gondii* infection disrupts this balance via strongly inducing the polarization of dMφ towards the pro-inflammatory M1 phenotype, thereby triggering a severe inflammatory immune response. Studies have shown that in BMDMs, the matrix antigen protein 1 (MAG1) from *T. gondii* (ME49 strain) is secreted into the host cytosol and suppresses IL-1β transcription [42]. In contrast, BMDMs infected with MAG1-deficient parasites exhibit increased IL-1β release mediated by GRA15 [42]. This suggests that while *T. gondii* disrupts host immune balance by secreting MAG1 to limit excessive inflammation, the presence of GRA15 suppresses key anti-inflammatory molecules [43]. Interestingly, Trem2 is a critical anti-inflammatory regulator expressed on dMφ, and its dysregulation exacerbates inflammation [44]. Our research has also determined that in *T. gondii*-infected placental tissue and *Tg*Ag-stimulated macrophages, *T. gondii* targets and suppresses the immune regulatory molecule Trem2, expressed on placental decidual macrophages, to disrupt placental defense. Single-cell sequencing demonstrated that *Trem2* knockout enhances pro-inflammatory macrophages in pancreatic ductal adenocarcinoma and exacerbates pathogenic inflammation [45]. Our previous findings establish Trem2 as a key defender of placental immune homeostasis against *T. gondii*, as its deficiency exacerbates APO [18]. Meanwhile, *Trem2*-deficient pregnant mouse models showed heightened susceptibility to *T. gondii* (RH strain) [18]. Therefore, Trem2 may regulate placental immunity through specific signaling pathways, and its deficiency exacerbates immune damage by promoting excessive inflammatory responses.

Trem2, which is a receptor for multiple ligands and suppresses inflammation, can also enhance the phagocytic activity of macrophages and autophagic-lysosomal activation [46,47,48]. Macrophage responses to pathogenic organisms and their molecular components (e.g., LPS) require actin cytoskeletal rearrangement [49]. Upon ligand binding to Trem2, phosphorylation of DAP12 initiates the Syk-PLCγ2 signaling axis. This induces calcium release and activates the PKC/mTOR pathway, ultimately driving actin cytoskeleton rearrangement [50]. However, the *T. gondii* surface antigen TgSAG1 can target the host protein S100A6, interfering with the interaction between S100A6 and vimentin [24]. This interference directly disrupts the dynamic reorganization of the cytoskeleton, potentially inhibiting phagocytosis-related structures. Notably, *T. gondii* can further suppress phagocytosis through autophagy-dependent lysosomal degradation or vacuolar disruption (e.g., mediated by GRA effector proteins) [51]. Therefore, we hypothesize that *T. gondii* disrupts autophagy-dependent lysosomal degradation and synergizes with other immunosuppressive mechanisms to antagonize the Trem2-mediated phagocytic clearance pathway, creating favorable conditions for its intracellular survival. In our study, through RNA-seq analysis of *Trem2*-overexpressing Raw264.7 macrophages, we found that MICAL1, a key contributor of autophagy, was ranked among the top 50 most significantly up-regulated genes. The correlation analysis further indicates a strong correlation between MICAL1 and Trem2. It is well known that the MICAL protein family has emerged as important redox enzymes regulating actin, influencing the properties of actin in vitro and in vivo [52]. Importantly, MICAL1 knockdown in oligodendrocyte cells could significantly reduce the levels of pro-autophagy factors (Beclin1 and LC3B), suggesting that MICAL1 can protect oligodendrocytes from oxidative injury via regulating autophagy [36]. RNA-seq analysis, molecular docking, fluorescence co-localization, and immunoprecipitation assays demonstrate that Trem2 directly interacts with MICAL1. Therefore, we identified MICAL1 as a critical target molecule within the Trem2-DAP12 signaling pathway, which conferred protection against *T. gondii* infection.

MICAL1 directly regulates cytoskeletal dynamics by disassembling F-actin and mediates ROS-dependent inflammatory signaling [52]. Based on the knowledge that the Trem2-DAP12 pathway activates PKC to promote actin reorganization, we preliminarily explored the potential association between Trem2 and MICAL1 using molecular docking simulations and fluorescence co-localization analysis. These studies revealed a high-affinity binding interaction between them. Recent studies have proposed that MICAL1 activation leads to upregulated ROS in HeLa cells and promotes autophagy [26,36]. Concurrently, MICAL1 depletion suppresses P-ERK expression, while *MICAL1* overexpression increases P-ERK nuclear translocation [26]. These findings suggest that MICAL1 may participate in regulating the immune response of macrophages through the ERK signaling pathway, and that *T. gondii* infection may disrupt this balance by interfering with the Trem2-MICAL1-P-ERK pathway.

In macrophages, P-ERK, as a downstream effector molecule of the Trem2-MICAL1 pathway, dynamically regulates the balance of immune responses through its phosphorylation levels [53,54]. MICAL1-induced breast cancer cell invasion relies on the ROS/PI3K/Akt signaling pathway, and inhibition of ROS activation can downregulate the MAPK/ERK pathway [26,55,56]. Both our animal and cell culture models revealed that *T. gondii* infection suppresses ERK phosphorylation, while overexpression of *Trem2*/*MICAL1* preserves P-ERK levels. Notably, in *Trem2*-knockout mice, *T. gondii* infection resulted in a more severe downregulation of P-ERK and enhanced APO, indicating that the Trem2-MICAL1 axis acts as a positive upstream regulator of P-ERK. These findings suggest that *T. gondii* may target and inhibit the Trem2-MICAL1 signaling pathway. This inhibition consequently suppresses the activity of its key downstream effector molecule, P-ERK, ultimately blocking the ROS activation pathway [57]. This suppression of the Trem2-MICAL1-P-ERK axis represents a key mechanism by which *T. gondii* weakens host immune defenses and induces APO.

In summary, we identified MICAL1 as a critical target molecule within the Trem2 signaling pathway and unraveled a critical protective role of the Trem2-MICAL1-P-ERK immunoregulatory signaling pathway in *T. gondii*-mediated APO. *T. gondii* disrupts the integrity of this signaling pathway by targeting Trem2 and impairing its interaction with MICAL1. This disruption leads to the loss of downstream P-ERK activity, ultimately compromising macrophage phagocytic clearance capacity and immunoregulatory functions.

## 5. Limitations of the Study

While this study establishes that Trem2 deficiency exacerbates APO after *T. gondii* infection, the functional impact of Trem2 reconstitution or knock-in remains undetermined. Moreover, the relevance of these findings to humans is unclear, as evidence is primarily derived from animal models, and a lack of clinical data hampers the translation of Trem2’s role in human pregnancy.

## Data Availability

The study data are openly accessible in Mendeley Data at https://data.mendeley.com/datasets/pvgdgx59tz/1, accessed on 1 September 2025.
